# Effects of Sarpogrelate Hydrochloride in Combination with Aspirin on Blood Viscosity and Hemorheological Parameters in Patients with Peripheral and Coronary Artery Disease: A Randomized Controlled Trial

**DOI:** 10.3390/ph19091410

**Published:** 2026-09-07

**Authors:** Yuran Ahn, Jaehyuk Jang, Seonghyeon Bu, Nay Aung, Hyo-Suk Ahn, Keun-Sang Yum

**Affiliations:** 1 Division of Cardiology, Department of Internal Medicine, Uijeongbu St. Mary’s Hospital, College of Medicine, The Catholic University of Korea, Seoul 06591, Republic of Korea; niceayr@naver.com (Y.A.); let87@naver.com (J.J.); buseonghyeon@gmail.com (S.B.); 2 Cardiovascular Research Institute for Intractable Disease, College of Medicine, The Catholic University of Korea, 222 Banpodaero, Seochogu, Seoul 06591, Republic of Korea; 3 William Harvey Research Institute, Barts and The London Faculty of Medicine and Dentistry, Queen Mary University of London, London E1 4NS, UK; 4 Department of Family Medicine, Uijeongbu St. Mary’s Hospital, College of Medicine, The Catholic University of Korea, Seoul 06591, Republic of Korea

**Keywords:** sarpogrelate hydrochloride, aspirin, blood viscosity, hemorheology, peripheral arterial disease, coronary artery disease

## Abstract

**Background/Objectives:** Hemorheological abnormalities, including increased blood viscosity and impaired red blood cell (RBC) deformability, contribute to poor vascular outcomes in patients with atherosclerotic diseases. Although sarpogrelate has shown vascular benefits, its additive effect with aspirin on blood viscosity remains unclear. This study aimed to evaluate whether adding sarpogrelate hydrochloride to aspirin improves blood viscosity and hemorheological parameters compared with aspirin monotherapy in patients with peripheral arterial disease (PAD) and coronary artery disease (CAD). **Methods:** This single-center, randomized, open-label trial evaluated sarpogrelate hydrochloride (300 mg/day) plus aspirin (100 mg/day) versus aspirin monotherapy over 12 weeks in patients with PAD and CAD. Patients were assigned using a computer-generated sequence. The primary analysis used the full analysis set. The primary outcome was change in systolic and diastolic blood viscosity. Secondary outcomes included RBC deformability, aggregation index, critical shear stress, flow-mediated dilation (FMD), metabolic parameters, and quality of life. FMD was performed using standardized protocols with blinded assessment. **Results:** Sixty-eight patients were randomized (34 per group), of whom 61 were included in the full analysis set (experimental group, *n* = 31; control group, *n* = 30). At week 12, systolic blood viscosity remained stable in the experimental group (4.51 → 4.49 cP) but increased in the control group (4.67 → 4.87 cP). The unadjusted mean between-group differences in change from baseline (experimental minus control) were −0.23 cP (95% CI, −0.66 to 0.20) for systolic blood viscosity and −0.55 cP (95% CI, −2.03 to 0.93) for diastolic blood viscosity; the corresponding longitudinal between-group *p*-values from the MMRM analyses were 0.9481 and 0.9630, respectively. FMD showed no significant between-group difference at week 12 (unadjusted mean difference, −0.60%; 95% CI, −2.05 to 0.85; MMRM *p* = 0.7727). Patient-reported outcomes (SF-36 and visual analog scale scores) did not differ significantly between groups. **Conclusions:** In patients with PAD and CAD, no statistically significant additional hemorheological benefit of sarpogrelate combined with aspirin was demonstrated compared with aspirin monotherapy.

## 1. Introduction

Coronary artery disease (CAD) and peripheral arterial disease (PAD) are common manifestations of systemic atherosclerosis that contribute to increased cardiovascular morbidity and mortality [1]. Impaired blood rheology, including increased whole blood viscosity, reduced red blood cell (RBC) deformability, and enhanced aggregation, plays a critical role in the progression of atherosclerotic vascular diseases and microcirculatory dysfunction [2,3]. These hemorheological abnormalities are associated with impaired tissue perfusion and increased thrombotic risk, particularly in patients with PAD and CAD [4,5].

Antiplatelet therapy with aspirin remains the cornerstone of secondary prevention in atherosclerotic cardiovascular disease [6,7]. However, owing to its limited effect on blood rheology, combination strategies with other agents are being explored [8,9]. Sarpogrelate hydrochloride is a selective 5-hydroxytryptamine 2A (5-HT_2A_) receptor antagonist. 5-HT is mainly stored in platelets and is released when the vascular endothelium is injured. 5-HT2A receptors are expressed on vascular smooth muscle cells and platelets. Several mechanisms of action of sarpogrelate have been proposed, including the selective blockade of 5-HT_2A_ receptors on vascular smooth muscle cells. Sarpogrelate is thought to inhibit serotonin-mediated vasoconstriction, platelet aggregation, and vascular smooth muscle proliferation [10,11]. In addition to its established effects on platelets and the vascular wall, sarpogrelate has been reported to improve erythrocyte deformability and reduce shear stress-induced hemolysis in experimental models, suggesting potential effects on hemorheological properties. Several studies have reported the potential of sarpogrelate to improve microcirculation, endothelial function, and clinical outcomes in patients with peripheral and coronary vascular disorders [12,13,14].

Despite these promising findings, few studies have evaluated the effects of sarpogrelate in combination with aspirin, particularly in the context of hemorheological parameters such as blood viscosity, RBC deformability, and aggregation. Whether the addition of sarpogrelate to standard antiplatelet therapy confers further benefits in patients with stable PAD and CAD remains unclear. This study aimed to investigate the effects of combining sarpogrelate hydrochloride with aspirin, compared with aspirin monotherapy, on blood viscosity and other hemorheological parameters in patients with both PAD and CAD. The study sought to determine whether sarpogrelate offers additional therapeutic value by improving hemorheological parameters beyond what is achieved with aspirin alone.

## 2. Results

### 2.1. Baseline Characteristics

The baseline characteristics of the participants are summarized in Table 1. The mean age of the participants was approximately 66 years, with a higher proportion of males in both groups. The body mass index (BMI) was comparable between the groups, and fasting blood glucose levels were lower in the experimental group than in the control group. A significant difference in smoking history was observed, with a higher percentage of past smokers in the experimental group. High-sensitivity C-reactive protein (hs-CRP) levels were similar between the two groups.

### 2.2. Changes in Blood Viscosity

The primary endpoint of the study was the change in blood viscosity from baseline to 12 weeks. Table 2 presents the changes in systolic and diastolic blood viscosity. At baseline, systolic blood viscosity was comparable between the two groups (control group, 4.67 cP; experimental group, 4.51 cP). After 12 weeks, systolic blood viscosity slightly increased in the control group (4.87 cP) but remained stable in the experimental group (4.49 cP). MMRM analysis demonstrated no significant treatment-by-visit interaction for systolic blood viscosity (*p* = 0.9481). The mean change from baseline was 0.21cP in the control group and −0.02 cP in the experimental group.

Similarly, no significant treatment-by-visit interaction was observed for diastolic blood viscosity (*p* = 0.9630). The mean change from baseline was 0.31 cP in the control group and −0.24 cP in the experimental group. Based on the observed changes from baseline, the unadjusted mean between-group difference (experimental minus control) at week 12 was −0.23 cP (95% CI, −0.66 to 0.20) for systolic blood viscosity and −0.55 cP (95% CI, −2.03 to 0.93) for diastolic blood viscosity.

### 2.3. Secondary Outcomes

Secondary outcome results are presented in Tables S1–S8. At week 4, no significant differences in systolic or diastolic blood viscosity were observed between the groups (Table S1). The mean change in systolic viscosity was −0.04 ± 0.53 cP in the experimental group and −0.05 ± 0.78 cP in the control group. Diastolic viscosity changes were also not significant (*p* = 0.4930).

MMRM analyses did not find any significant between-group differences over time in RBC deformability, aggregation index, critical shear stress, or FMD (Tables S2 and S3). At week 4, the mean change in FMD from baseline was 0.10 ± 3.00% in the experimental group and 0.68 ± 1.12% in the control group, with no significant between-group difference (*p* = 0.4517). The unadjusted mean between-group difference (experimental minus control) was −0.58% (95% CI, −1.75 to 0.59). The within-group increase at week 4 was significant in the control group (*p* = 0.0017) but not in the experimental group (*p* = 0.0985). At week 12, the mean changes from baseline were 0.65 ± 2.98% and 1.25 ± 2.68% in the experimental and control groups, respectively, with no significant between-group difference (*p* = 0.7727). The unadjusted mean between-group difference was −0.60% (95% CI, −2.05 to 0.85). At week 12, significant within-group increases from baseline were observed in both the experimental (*p* = 0.0467) and control (*p* = 0.0173) groups. The tissue oxygen delivery index (tODI), lipid profiles, and homeostasis model assessment of insulin resistance (HOMA-IR) did not show meaningful changes from baseline or differences between groups (Tables S4, S5, and S7, respectively). Fasting blood glucose levels tended to decrease in both groups, with significant within-group reductions observed in the control group at both time points (Table S6). hs-CRP levels decreased significantly in the control group at week 12 (*p* = 0.0351), but no change was observed in the experimental group (Table S8). Additionally, no significant differences were observed between the two groups in patient-reported outcomes, including SF-36 domain scores and visual analog scale (VAS) ratings.

### 2.4. Adverse Events

The safety profiles of both treatment regimens were evaluated in the safety analysis set, which consisted of 34 participants in each treatment group (68 participants in total). The safety endpoints of this study included the incidence of adverse events, such as minor bruising, serious hemorrhages, intracranial bleeding, upper and lower gastrointestinal bleeding, and allergic reactions. Discontinuations were due to adverse events, which were assessed based on physical examinations, vital signs, and clinical laboratory tests. The most frequently reported adverse events by organ system were gastrointestinal disorders (4 patients, 5.88%); nervous system disorders (4 patients, 5.88%); respiratory, thoracic, and mediastinal disorders (3 patients, 4.41%); and renal and urinary disorders (2 patients, 2.94%). In the experimental group, gastrointestinal disorders were the most common events (3 patients, 8.8%), followed by nervous system disorders (2 patients, 5.8%) and respiratory, thoracic, and mediastinal disorders (2 patients, 5.8%). In the control group, nervous system disorders (2 patients, 5.8%) and renal and urinary disorders (2 patients, 5.8%) were the most frequently reported adverse events (Table S9). As shown in Table 3, the proportion of patients who experienced adverse events was similar between the groups: 26.47% and 23.53% of patients in the experimental and control groups, respectively, experienced at least one adverse event. Drug-related adverse events were observed in 5.88% of the patients in each group. The rate of discontinuation owing to adverse events was low and comparable between the groups. No serious adverse events or serious adverse drug reactions occurred in either group during the study period.

## 3. Discussion

This study evaluated the effects of sarpogrelate hydrochloride in combination with aspirin compared with those of aspirin monotherapy on blood viscosity and related hemorheological parameters in patients with both PAD and CAD. The primary outcome, blood viscosity at week 12, was not significantly different between the two groups. Similarly, the secondary outcomes, including changes in RBC deformability, aggregation index, critical shear stress, and FMD, were not significantly different between the groups. These findings indicate that there was no statistically significant additional benefit of sarpogrelate hydrochloride when combined with aspirin in terms of modifying blood viscosity or improving vascular function compared with aspirin alone over the 12-week treatment period. However, although the achieved sample size exceeded the prospectively calculated minimum requirement, the possibility of insufficient statistical power to detect small but clinically relevant treatment effects cannot be excluded, particularly given the substantial inter-individual variability and the mild baseline disease status with near-normal hemorheological parameters. In addition, the relatively mild disease status and near-normal baseline hemorheological profiles of the enrolled participants may have limited the potential for measurable improvement, resulting in a possible ceiling effect. Previous studies have shown that sarpogrelate reduces platelet aggregation, improves blood flow, and may have beneficial effects on endothelial function [15,16,17]. Serotonin plays a significant role in the regulation of vascular tone, platelet aggregation, and microvascular circulation [18,19,20]. Several studies have demonstrated that serotonin stimulates vascular smooth muscle cell proliferation [20,21,22]. Sarpogrelate blocks serotonin-mediated effects, thereby improving blood flow in both the macrovascular and microvascular systems [10,13,23]. Clinical trials have demonstrated the benefits of sarpogrelate in patients with PAD, diabetic microangiopathy, and coronary microvascular dysfunction [12,24,25]. However, whole blood viscosity is largely determined by the hematocrit, plasma viscosity, and erythrocyte deformability and aggregation, which may not be directly or substantially affected by serotonin signaling pathways.

Despite the promising pharmacological effects of sarpogrelate, this study did not demonstrate a statistically significant additional benefit of sarpogrelate when combined with aspirin. This discrepancy may be explained by several factors. First, because all participants were already receiving aspirin, which exerts antiplatelet and mild anti-inflammatory effects, the additive benefit of sarpogrelate may have been masked. The effects of aspirin on platelet function and vascular tone may have limited the ability to detect additional hemorheological improvements induced by sarpogrelate. Second, although the vascular and microcirculatory benefits of sarpogrelate are well established, its direct effects on blood viscosity remain unclear. While some antiplatelet or rheologic agents, such as clopidogrel [26,27] or ticagrelor [28,29], have been associated with reductions in blood viscosity, others, including cilostazol [30] and ticlopidine [31], have not demonstrated such effects. Blood viscosity is influenced by multiple factors, including hematocrit, plasma viscosity, and RBC deformability and aggregation, which may not be substantially affected by serotonin receptor antagonism alone. With respect to endothelial function, FMD increased significantly only in the aspirin monotherapy group at week 4; however, the between-group difference in FMD change was not significant. By week 12, significant within-group increases were observed in both treatment groups, while the between-group difference remained non-significant. Therefore, the observed pattern does not provide evidence that sarpogrelate attenuated the effect of aspirin on endothelial function. Nevertheless, given the limited sample size and wide confidence intervals, a small differential effect cannot be excluded.

Additionally, differences in patient populations may contribute to these contrasting findings. Previous studies often enrolled patients with more advanced or symptomatic vascular disease, whereas the participants in this study had mild or stable disease and near-normal baseline blood viscosity. Yum et al. reported a significant reduction in blood viscosity after sarpogrelate treatment in patients with higher baseline viscosity. In contrast, the present study included patients with mild disease and near-normal hemorheological profiles, which may have limited the potential for measurable change. This study has the strength of being a randomized controlled clinical trial that prospectively evaluated hemorheological and vascular parameters using standardized methods. However, the study has several limitations. First, the treatment duration of 12 weeks may have been insufficient to observe meaningful changes in blood viscosity or endothelial function, particularly in a population with stable disease. Second, the study included patients with both PAD and CAD; however, most patients had mild disease and near-normal baseline hemorheological values. This may have limited the potential for detectable therapeutic effects. Third, the single-center design and inclusion of only Korean participants may limit the generalizability of our findings to other populations and healthcare settings. In addition, the study eligibility criteria may have selected a relatively homogeneous population with stable PAD and CAD, potentially limiting the applicability of these findings to patients with more severe or heterogeneous vascular disease. The sample size, while adequate for detecting moderate differences, may have been underpowered to identify small but clinically relevant effects on secondary outcomes. In addition, substantial inter-individual variability was observed in some hemorheological measurements, particularly diastolic blood viscosity, for which the standard deviation of the change was relatively large compared with the mean change. This variability may have increased uncertainty in the treatment effect estimates and further limited the statistical sensitivity to detect small treatment effects. Given the small sample size and real-world nature of this study, baseline imbalances were observed between the groups, particularly in smoking status. These factors are known to influence vascular function, inflammatory markers, and hemorheological parameters. Smoking, in particular, affects blood viscosity, erythrocyte deformability, and endothelial function. The baseline imbalance in smoking status between the groups may therefore have influenced the observed hemorheological and vascular outcomes. Consequently, residual confounding cannot be excluded, and the results should be interpreted with caution. The open-label design represents an important limitation of this study. Because participants and treating physicians were aware of treatment allocation, subjective outcomes, including SF-36 and VAS scores, may have been influenced by expectation and performance bias, while adverse event reporting may have been susceptible to reporting bias. Although FMD was assessed using a standardized protocol with blinded assessment and the primary endpoint of blood viscosity was an objectively measured laboratory parameter, the potential influence of the open-label design cannot be completely excluded. A double-blind, placebo-controlled design would have provided a more rigorous assessment of treatment effects and should be considered in future studies.

## 4. Materials and Methods

### 4.1. Study Design and Participants

This was a prospective, randomized, parallel-group, open-label, single-center study. Eligible participants were adults diagnosed with PAD and CAD who met the study inclusion criteria and provided written informed consent. Patients were randomly assigned to receive either aspirin 100 mg (Aspirin Protect Tablets 100 mg; Bayer Korea, Seoul, Republic of Korea) monotherapy (control group) or aspirin 100 mg plus sarpogrelate hydrochloride 300 mg (Anplag SR Tablets 300 mg; Yuhan Corporation, Seoul, Republic of Korea) (experimental group) once daily for a duration of 12 weeks.

Patients were eligible for inclusion if they were aged ≥19 years and had a confirmed diagnosis of CAD with coronary artery stenosis of 10–75% confirmed by coronary angiography or coronary computed tomography angiography. Participants were also required to have a diagnosis of PAD or present with symptoms indicative of PAD, including ischemic pain, intermittent claudication, ulcers, or other manifestations of vascular insufficiency (Table 1). A broad spectrum of patients with CAD and PAD was included to reflect real-world clinical practice. Accordingly, patients with mild-to-moderate disease were also included. Although this approach improved the generalizability of the study findings, some participants may have had subtle baseline hemorheological alterations that could have reduced the observable magnitude of changes in blood viscosity.

The exclusion criteria included patients who were scheduled for coronary or cerebrovascular revascularization procedures, those who had used aspirin within 2 weeks before randomization, and those receiving anticoagulants or antiplatelet agents other than aspirin. Individuals with severe renal impairment (estimated glomerular filtration rate < 30 mL/min/1.73 m^2^), hemoglobin levels below 13 g/dL (or below 12 g/dL for females), platelet counts below 60,000/µL, or active bleeding disorders were also excluded. Additional exclusion criteria included a history of myocardial infarction, unstable angina, stroke, or transient ischemic attack within the past 6 months; pregnancy or lactation; and participation in other investigational drug trials.

**Figure 1:**
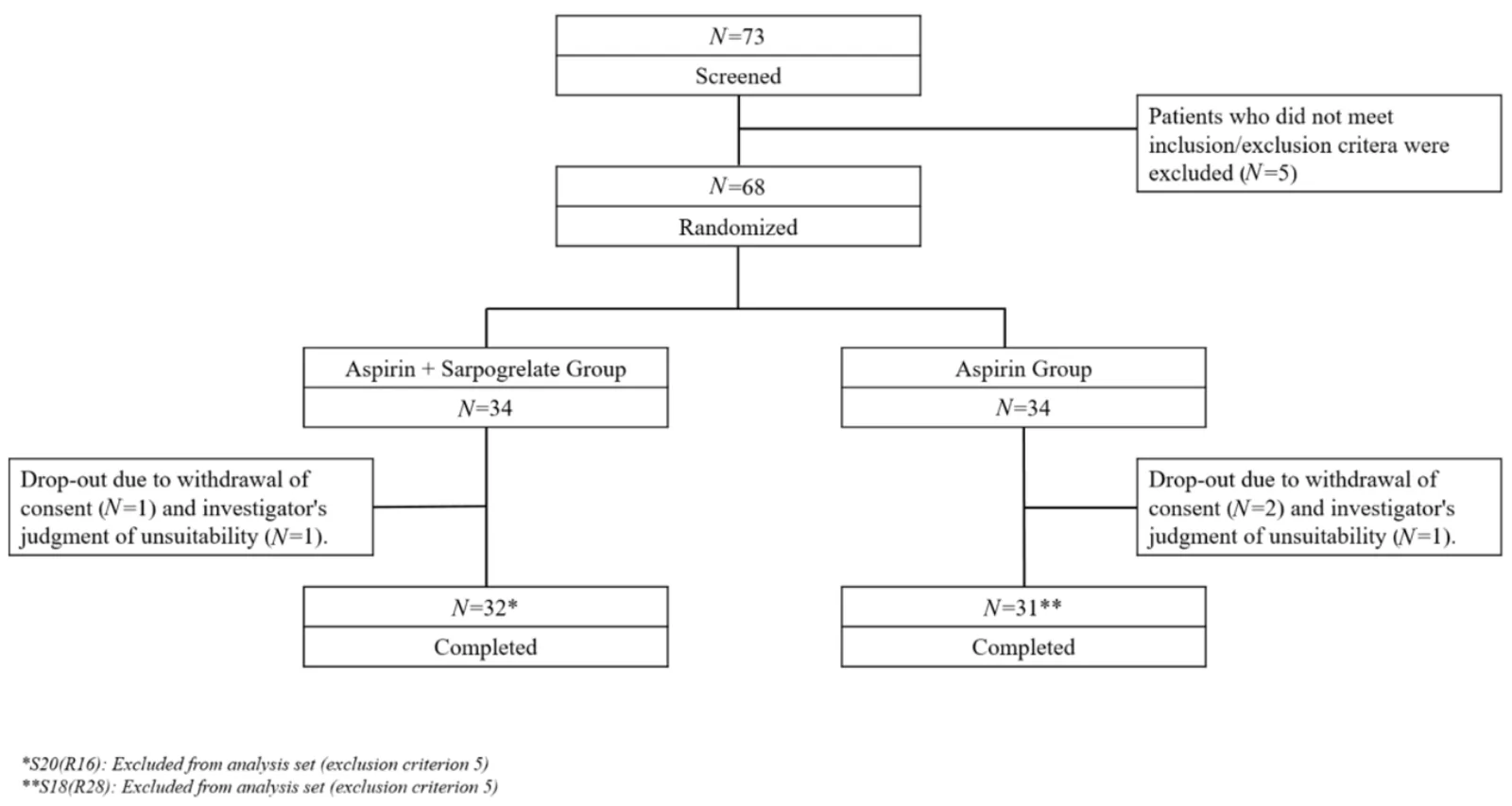
Flowchart of patient enrollment, randomization, and study progress.

The study enrolled 68 patients diagnosed with PAD and CAD. The participants were randomly assigned to two groups: 34 patients received aspirin 100 mg monotherapy (control group), and 34 patients received a combination of aspirin 100 mg and sarpogrelate hydrochloride 300 mg (experimental group). After accounting for dropouts, 32 and 31 patients in the experimental and control groups, respectively, completed the study. Of these, 31 participants in the experimental group and 30 participants in the control group were included in both the FAS and PPS. The safety analysis set comprised all 68 randomized participants (34 per group). The selection process is illustrated in Figure 1. Both FMD image acquisition and subsequent image analysis were performed by assessors who were blinded to treatment allocation. During operator training, measurements were performed using the same ultrasound machine(Vivid E95; GE HealthCare, Seoul, Republic of Korea) to ensure consistency in measurement values.

### 4.2. Randomization and Intervention

**Figure 2:**
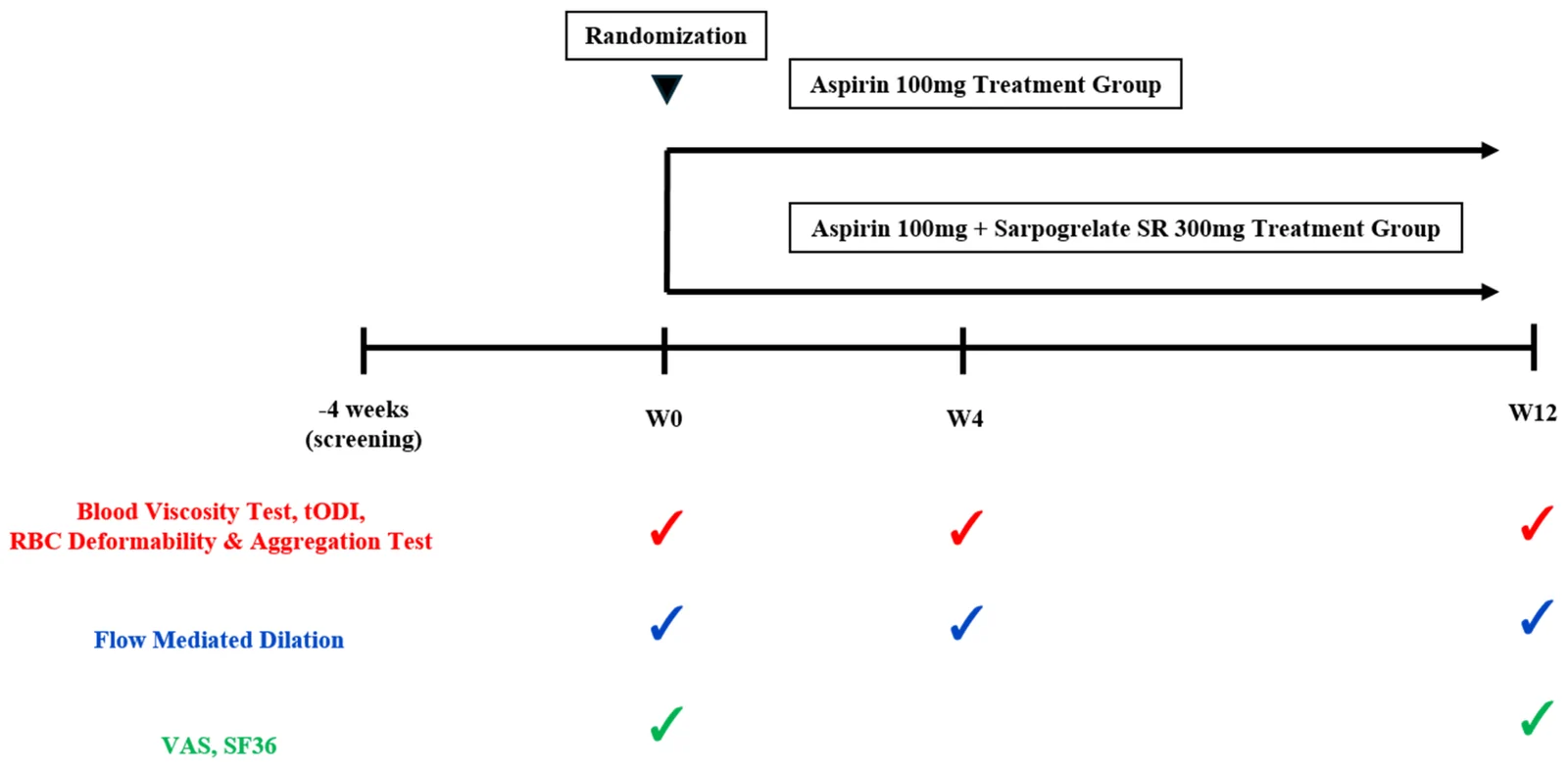
Study design and assessment schedule. Checkmarks (✓) indicate the scheduled assessment time points.

An overview of the study process is shown in Figure 2. The study included a 12-month recruitment period comprising a 4-week screening phase and a 12-week treatment phase. Participants were randomly assigned in a 1:1 ratio to the experimental or control group using block randomization with a block size of four. The randomization sequence was independently generated by a statistician who was not otherwise involved in the study to minimize potential allocation bias. Participants were assigned according to the prespecified randomization schedule, and randomization numbers were confirmed through the electronic data capture (EDC) system. Randomization was performed using a centralized system to maintain allocation concealment.

The control group received aspirin 100 mg once daily for 12 weeks, whereas the experimental group received a combination of aspirin 100 mg and sarpogrelate hydrochloride 300 mg once daily for the same duration. To promote adherence, pill counts and self-reported compliance were assessed during follow-up visits. The participants were instructed to maintain at least 80% adherence. Study evaluations were conducted at weeks 4 and 12.

### 4.3. Outcome Measures

The primary outcome was the change in whole blood viscosity from baseline to week 12, assessed under both systolic and diastolic shear conditions. Systolic and diastolic blood viscosity were analyzed separately as two measures of the prespecified primary outcome. Secondary outcomes included changes in erythrocyte deformability, erythrocyte aggregation, FMD, tODI, lipid profiles, fasting blood glucose, HOMA-IR, and hs-CRP levels. Changes in blood viscosity from baseline to 4 weeks were also measured. Additionally, patient-reported quality of life assessments, including SF-36 and VAS scores, were performed at baseline and week 12. Adverse and serious adverse events were recorded throughout the study period to assess safety.

Blood samples were collected at baseline, 4 weeks, and 12 weeks to measure primary and secondary biomarkers. Blood viscosity was determined using the scanning capillary technique RheoSCAN-AnD300 (RheoMeditech Inc., Seoul, Republic of Korea). Systolic blood viscosity was defined as whole-blood viscosity measured at a high shear rate of 300 s^−1^, whereas diastolic blood viscosity was defined as whole-blood viscosity measured at a low shear rate of 5 s^−1^. Diastolic blood viscosity used for the tODI calculation was measured at 37.5 °C. FMD was evaluated using high-resolution ultrasound imaging of the brachial artery (Vivid E95; GE HealthCare, Seoul, Republic of Korea), following established protocols for assessing endothelial function. The tODI was calculated as the ratio of hematocrit (%) to diastolic blood viscosity (DBV) measured at a low shear rate of 5 s^−1^ and a temperature of 37.5 °C. Standard laboratory assays were performed to measure lipid profiles, fasting glucose, HOMA-IR, and hs-CRP.

### 4.4. Statistical Analysis

At the time of study design, no previous clinical trial had evaluated the effect of aspirin combined with sarpogrelate hydrochloride on blood viscosity. Therefore the sample size was determined based on the most comparable published study evaluating aspirin plus dipyridamole by Rosenson et al. [8]. Assuming a two-sided significance level of 0.05 and a statistical power of 80%, the required sample size was estimated to be 27 participants per group based on systolic blood viscosity and 26 participants per group based on diastolic blood viscosity. After allowing for an anticipated dropout rate of 20%, the final target sample size was determined to be 68 participants (34 per group). All efficacy analyses were based on the full analysis set (FAS). The FAS comprised 31 participants in the experimental group and 30 participants in the control group, including participants who received at least one dose of the investigational product and had available data for at least one efficacy endpoint. One participant in each group was excluded from the efficacy analysis because a prespecified exclusion criterion (history of cerebrovascular or cardiovascular complications within the previous 6 months) was identified after randomization. The per-protocol set was also defined; however, as both sets consisted of the same patient population and yielded identical results (experimental group, *n* = 31; control group, *n* = 30), only the FAS results are reported. In the aspirin plus sarpogrelate group, two participants discontinued the study (one withdrew consent, and one was deemed unsuitable by the investigators). In the aspirin-only group, three participants discontinued the study (two withdrew consent, and one was deemed unsuitable by the investigators). Safety analyses were performed using the safety analysis set, which included all randomized participants who received at least one dose of the study medication and had post-treatment safety follow-up. Continuous variables are summarized as mean ± standard deviation (SD), median, and range (minimum to maximum). Between-group comparisons were conducted using the independent *t*-test or Wilcoxon rank-sum test depending on the data distribution. For efficacy analyses, repeated measurements were analyzed using a mixed-effects model for repeated measures (MMRM), with treatment group, visit, and treatment-by-visit interaction included as fixed effects. An unstructured (UN) covariance structure was used for the repeated measurements. The reported MMRM *p*-values correspond to the treatment-by-visit interaction. As prespecified in the statistical analysis plan (SAP), missing values were not imputed, and analyses were performed using the available observed data. The normality of the data distribution was assessed using the Shapiro–Wilk test. Statistical significance was set at *p* < 0.05. Data were analyzed using SAS software, version 9.4 (SAS Institute Inc. Cary, NC, USA: Ver. 9.4).

## 5. Conclusions

In this randomized controlled trial involving patients with PAD and CAD, combination therapy with sarpogrelate hydrochloride and aspirin did not demonstrate superior efficacy to aspirin monotherapy in improving blood viscosity or hemorheological parameters over a 12-week period. Secondary outcomes, including RBC deformability, aggregation, endothelial function, and metabolic biomarkers, showed no significant between-group differences. Further studies targeting high-risk populations or those with elevated baseline viscosity may help clarify the potential role of sarpogrelate in vascular therapy.

## Figures and Tables

**Table 1:** Baseline characteristics of study participants.

Category	Characteristic	Experimental Group (*N* = 34)	Control Group (*N* = 34)	*p*-Value
Sex n (%)	Total	34 (100.00)	34 (100.00)	-
	Male	25 (73.53)	26 (76.47)	0.7794
	Female	9 (26.47)	8 (23.53)	-
Age (years)	Mean ± SD	65.53 ± 6.45	66.65 ± 9.75	0.5796 *
	Median	66	68	-
	Min, Max	51.00, 76.00	34.00, 85.00	-
Age Group n (%)	10s	0 (0.00)	0 (0.00)	-
	20s	0 (0.00)	0 (0.00)	-
	30s	0 (0.00)	1 (2.94)	-
	40s	0 (0.00)	0 (0.00)	-
	50s	6 (17.65)	7 (20.59)	-
	60s	20 (58.82)	11 (32.35)	-
	70s and older	8 (23.53)	15 (44.12)	-
Pregnancy/Lactation n (%) †	Yes	0 (0.00)	0 (0.00)	-
	No	9 (26.47)	8 (23.53)	-
Smoking Status n (%)	No past smoking	8 (23.53)	13 (38.24)	0.0244
	Current smoker	4 (11.76)	10 (29.41)	-
	Former smoker	22 (64.71)	11 (32.35)	-
Cigarettes per day	Mean ± SD	16.92 ± 12.91	25.76 ± 20.88	0.0498 **
	Median	13	12	-
	Min, Max	2.00, 60.00	3.00, 100.00	-
Alcohol Consumption n (%)	No	11 (32.35)	9 (26.47)	0.8584
	Current drinker	18 (52.94)	20 (58.82)	-
	Heavy drinker	5 (14.71)	5 (14.71)	-
Alcohol intake (g)	Mean ± SD	84.35 ± 45.31	98.20 ± 99.18	0.7178 **
	Median	70	70	-
	Min, Max	20.00, 140.00	10.00, 420.00	-
Height (cm)	Mean ± SD	164.41 ± 6.85	162.85 ± 8.62	0.4112 *
	Median	165.5	162	-
	Min, Max	150.00, 177.00	138.00, 180.00	-
Weight (kg)	Mean ± SD	70.56 ± 8.16	68.95 ± 13.26	0.5487 *
	Median	72.8	69.9	-
	Min, Max	55.00, 84.00	48.00, 98.50	-
BMI (kg/m^2^)	Mean ± SD	26.11 ± 2.82	25.85 ± 3.64	0.7415 *

† Pregnancy/lactation status was assessed only in female participants (experimental group, n = 9; control group, *n* = 8). * Independent *t*-test, ** Wilcoxon rank-sum test. -, not applicable.

**Table 2:** Change in blood viscosity from baseline to week 12.

Parameter	Aspirin + Sarpogrelate Group (*n* = 31)	Aspirin Group (*n* = 30)	*p*-Value **	MMRM *p*-Value ‡
**Systolic Blood Viscosity (cP)**				
Baseline			0.4432	
mean ± SD	4.51 ± 0.72	4.67 ± 0.75		
median	4.30	4.55		
min–max	3.40–6.40	3.70–7.00		
Week 12			0.2717	
mean ± SD	4.49 ± 0.73	4.87 ± 1.06		
median	4.40	4.55		
min–max	3.60–6.30	3.60–7.10		
Change from baseline			0.3668	0.9481
mean ± SD	−0.02 ± 0.79	0.21 ± 0.88		
median	0.00	0.10		
min–max	−2.10–2.90	−1.50–2.30		
Week 12-Baseline *p*-value	0.6812 ††	0.2105 †		
**Diastolic Blood Viscosity (cP)**				
Baseline			0.5834	
mean ± SD	13.85 ± 2.29	14.23 ± 2.44		
median	13.20	13.70		
min–max	9.50–18.20	10.40–20.80		
Week 12			0.1593	
mean ± SD	13.61 ± 2.61	14.54 ± 2.83		
median	13.40	13.75		
min–max	10.40–21.80	10.10–20.20		
Change from baseline			0.7400	0.9630
mean ± SD	−0.24 ± 3.34	0.31 ± 2.35		
median	−0.30	−0.55		
min–max	−6.60–12.30	−3.50–5.90		
Week 12-Baseline *p*-value	0.4498 ††	0.9122 ††		

SD = Standard Deviation, ** Wilcoxon rank sum test, † paired *t*-test, †† Wilcoxon signed-rank test, ‡ *p* values were obtained using a mixed-effects model for repeated measures (MMRM).

**Table 3:** Summary of adverse events.

Parameter	Aspirin + Sarpogrelate Group (*N* = 34)	Aspirin Group (*N* = 34)	*p*-Value
Any AE, n (%)	9 (26.47)	8 (23.53)	0.7794
Drug-related AE, n (%)	2 (5.88)	2 (5.88)	>0.05
AE leading to discontinuation, n (%)	2 (5.88)	1 (2.94)	>0.05

## Data Availability

The data presented in this study are available on request from the corresponding author due to ethical restrictions, as the informed consent obtained from the study participants did not include consent for sharing research data with third parties.

## Supplementary Materials

The following supporting information can be downloaded at: https://www.mdpi.com/article/10.3390/ph19091410/s1, Table S1: Changes in systolic/diastolic blood viscosity from baseline to week 4; Table S2: Changes in RBC deformability and aggregation from baseline to weeks 4 and 12; Table S3: Changes in FMD from baseline to weeks 4 and 12; Table S4: Changes in the tODI from baseline to weeks 4 and 12; Table S5: Changes in lipid profile from baseline to weeks 4 and 12; Table S6: Changes in fasting blood glucose levels from baseline to weeks 4 and 12; Table S7: Changes in HOMA-IR from baseline to weeks 4 and 12; Table S8: Changes in hs-CRP levels from baseline to weeks 4 and 12; Table S9: Adverse Reaction Incidence Classified by Organ System.

## Abbreviations

The following abbreviations are used in this manuscript:

CAD	coronary artery disease
PAD	peripheral arterial disease
RBC	red blood cell
FMD	flow-mediated dilation
VAS	visual analogue scale
tODI	tissue oxygen delivery index
BMI	body mass index
5-HT	5-hydroxytryptamine
5-HT_2_A	5-hydroxytryptamine 2A receptor
cP	centipoise
SF-36	36-item short-form health survey

## References

[1] Libby P., Ridker P.M., Maseri A. (2002). Inflammation and Atherosclerosis. Circulation.

[2] Ikeda U. (2005). Inflammation and Coronary Artery Disease. Curr. Vasc. Pharmacol..

[3] Sloop G., Holsworth R.E., Weidman J.J., Cyr J.A. (2015). The Role of Chronic Hyperviscosity in Vascular Disease. Ther. Adv. Cardiovasc. Dis..

[4] Cekirdekci E.I., Bugan B. (2020). Whole Blood Viscosity in Microvascular Angina and Coronary Artery Disease: Significance and Utility. Rev. Port. Cardiol..

[5] Baskurt O.K., Meiselman H.J. (2023). Blood Rheology and Hemodynamics. Semin. Thromb. Hemost..

[6] Baigent C., Sudlow C., Collins R., Peto R. (2002). Collaborative Meta-Analysis of Randomised Trials of Antiplatelet Therapy for Prevention of Death, Myocardial Infarction, and Stroke in High Risk Patients. Br. Med. J..

[7] Bhatt D.L., Topol E.J. (2003). Scientific and Therapeutic Advances in Antiplatelet Therapy. Nat. Rev. Drug Discov..

[8] Rosenson R.S. (2008). Treatment with Aspirin and Dipyridamole Is More Effective than Aspirin in Reducing Low Shear Blood Viscosity. Microcirculation.

[9] Rosenson R.S., Wolff D., Green D., Boss A.H., Kensey K.R. (2004). Aspirin. Aspirin Does Not Alter Native Blood Viscosity. J. Thromb. Haemost..

[10] Saini H.K., Takeda N., Goyal R.K., Kumamoto H., Arneja A.S., Dhalla N.S. (2004). Therapeutic Potentials of Sarpogrelate in Cardiovascular Disease. Cardiovasc. Drug Rev..

[11] Fernández-González J.F., García-Pedraza J.Á., Marín-Quílez A., Bastida J.M., Martín M.L., Morán A., García-Domingo M. (2022). Effect of Sarpogrelate Treatment on 5-HT Modulation of Vascular Sympathetic Innervation and Platelet Activity in Diabetic Rats. Biomed. Pharmacother..

[12] Satomura K., Takase B., Hamabe A., Ashida K., Hosaka H., Ohsuzu F., Kurita A. (2002). Sarpogrelate, a Specific 5HT2-Receptor Antagonist, Improves the Coronary Microcirculation in Coronary Artery Disease. Clin. Cardiol..

[13] Hayashi T., Sumi D., Matsui-Hirai H., Fukatsu A., Arockia J., Rani P.R., Kano H., Tsunekawa T., Iguchi A. (2003). Sarpogrelate HCl, a Selective 5-HT2A Antagonist, Retards the Progression of Atherosclerosis through a Novel Mechanism. Atherosclerosis.

[14] Lee D.H., Chun E.J., Hur J.H., Min S.H., Lee J.E., Oh T.J., Kim K.M., Jang H.C., Han S.J., Kang D.K. (2017). Effect of Sarpogrelate, a Selective 5-HT2A Receptor Antagonist, on Characteristics of Coronary Artery Disease in Patients with Type 2 Diabetes. Atherosclerosis.

[15] Hara H., Kitajima A., Shimada H., Tamao Y. (1991). Antithrombotic Effect of MCI-9042, a New Antiplatelet Agent on Experimental Thrombosis Models. Thromb. Haemost..

[16] Yamashita T., Kitamori K., Hashimoto M., Watanabe S., Giddings J.C., Yamamoto J. (2000). Conjunctive Effects of the 5HT2 Receptor Antagonist, Sarpogrelate, on Thrombolysis with Modified Tissue Plasminogen Activator in Different Laser-Induced Thrombosis Models. Haemostasis.

[17] Tamura K., Kanzaki T., Saito Y., Otabe M., Saito Y., Morisaki N. (1997). Serotonin (5-Hydroxytryptamine, 5-HT) Enhances Migration of Rat Aortic Smooth Muscle Cells through 5-HT2 Receptors. Atherosclerosis.

[18] Shimokawa H., Vanhoutte P.M. (1991). Angiographic Demonstration of Hyperconstriction Induced by Serotonin and Aggregating Platelets in Porcine Coronary Arteries with Regenerated Endothelium. J. Am. Coll. Cardiol..

[19] Vikenes K., Farstad M., Nordrehaug J.E. (1999). Serotonin Is Associated with Coronary Artery Disease and Cardiac Events. Circulation.

[20] Satoh K., Ozaki Y., Qi R., Yang L., Asazuma N., Yatomi Y., Kume S. (1996). Factors That Affect the Size of Platelet Aggregates in Epinephrine-Induced Activation: A Study Using the Particle Counting Method Based upon Light Scattering. Thromb. Res..

[21] Nemecek G.M., Coughlin S.R., Handley D.A., Moskowitz M.A. (1986). Stimulation of Aortic Smooth Muscle Cell Mitogenesis by Serotonin. Proc. Natl. Acad. Sci. USA.

[22] Lee S.L., Wang W.W., Moore B.J., Fanburg B.L. (1991). Dual Effect of Serotonin on Growth of Bovine Pulmonary Artery Smooth Muscle Cells in Culture. Circ. Res..

[23] Yum K.S., Kang S.G., Lee J.W., Cho Y.I. (2023). Effects of Sarpogrelate on Blood Viscosity. Microvasc. Res..

[24] Miyazaki M., Higashi Y., Goto C., Chayama K., Yoshizumi M., Sanada H., Orihashi K., Sueda T. (2007). Sarpogrelate Hydrochloride, a Selective 5-HT2A Antagonist, Improves Vascular Function in Patients with Peripheral Arterial Disease. J. Cardiovasc. Pharmacol..

[25] Hasegawa Y., Suehiro A., Higasa S., Namba M., Kakishita E. (2002). Enhancing Effect of Advanced Glycation End Products on Serotonin-Induced Platelet Aggregation in Patients with Diabetes Mellitus. Thromb. Res..

[26] Ciuffetti G., Lombardini R., Pirro M., Lupattelli G., Mannarino E. (2001). Clopidogrel: Hemorheological Effects in Subjects with Subclinical Atherosclerosis. Clin. Hemorheol. Microcirc..

[27] Koltai K., Papp J., Kenyeres P., Feher G., Tibold A., Alexy T., Marton Z., Kesmarky G., Toth K. (2014). Gender Differences in Hemorheological Parameters and in in Vitro Platelet Aggregation in Acetylsalicylic Acid and Clopidogrel Treated Vascular Patients. Biorheology.

[28] Rosenson R.S., Chen Q., Najera S.D., Lee M.L., Cho D.J. (2018). Ticagrelor and the Prevention of Microvascular Complications in Diabetes Patients with Lower Extremity Arterial Disease; Rationale and Design of the Hema-Kinesis Trial. Cardiovasc. Drugs Ther..

[29] Rosenson R.S., Chen Q., Najera S.D., Krishnan P., Lee M.L., Cho D.J. (2019). Ticagrelor Improves Blood Viscosity-Dependent Microcirculatory Flow in Patients with Lower Extremity Arterial Disease: The Hema-Kinesis Clinical Trial. Cardiovasc. Diabetol..

[30] Dawson D.L., Zheng Q., Worthy S.A., Charles B., Bradley D.V. (2002). Failure of Pentoxifylline or Cilostazol to Improve Blood and Plasma Viscosity, Fibrinogen, and Erythrocyte Deformability in Claudication. Angiology.

[31] Fagher B., Persson S., Persson G., Larsson H. (1993). Blood Viscosity During Long-Term Treatment with Ticlopidine in Patients with Intermittent Claudication. A Double-Blind Study. Angiology.

