# Supplementary material for: Effects of Sarpogrelate Hydrochloride in Combination with Aspirin on Blood Viscosity and Hemorheological Parameters in Patients with Peripheral and Coronary Artery Disease: A Randomized Controlled Trial
Source: Pharmaceuticals (Basel). 2026 Sep 7;19(9):1410. doi: 10.3390/ph19091410 (PMC13610731; doi:10.3390/ph19091410)
Supplement: Supplementary file 1 [file pharmaceuticals-19-01410-s001.zip › pharmaceuticals-4438930-supplementary.pdf]

Supplementary Materials

Table S1. Changes in systolic/diastolic blood viscosity from baseline to week 4.

Blood Viscosity	Visit	Measurement	Experimental Group	Control Group	*p*-value	*p*-value‡
Systolic	Visit 1	n (%)	31 (100.00)	30 (100.00)	-	0.9481
		mean±SD	4.51±0.72	4.67±0.75	0.4432**	
		Median	4.3	4.55	-	
		Min, Max	3.40, 6.40	3.70, 7.00	-	
	Visit 2	n (%)	31 (100.00)	30 (100.00)	-	
		mean±SD	4.47±0.70	4.62±0.78	0.1929**	
		Median	4.2	4.4	-	
		Min, Max	3.80, 6.10	3.20, 6.80	-	
	Change (Visit 2 - Visit 1)	n (%)	31 (100.00)	30 (100.00)	-	
		mean±SD	-0.04±0.53	-0.05±0.78	0.9481**	
		Median	-0.1	-0.2	-	
		Min, Max	-1.20, 1.40	-1.30, 2.30	0.7123	
Diastolic	Visit 1	n (%)	31 (100.00)	30 (100.00)	-	0.9630
		mean±SD	13.85±2.29	14.23±2.44	0.5834**	
		Median	13.2	13.7	-	
		Min, Max	9.50, 18.20	10.40, 20.80	-	
	Visit 2	n (%)	31 (100.00)	30 (100.00)	-	
		mean±SD	13.66±2.18	14.01±2.27	0.3785**	
		Median	13	13.2	-	
		Min, Max	10.90, 18.60	10.10, 19.40	-	
	Change (Visit 2 - Visit 1)	n (%)	31 (100.00)	30 (100.00)	-	
		mean±SD	-0.19±1.98	-0.22±2.42	0.4930**	
		Median	-0.3	-0.9	-	
		Min, Max	-3.90, 4.10	-3.10, 7.70	0.5962	

** Wilcoxon rank-sum test, #paired t-test, ##Wilcoxon signed-rank test, ‡ P values were obtained using a mixed-effects model for repeated measures (MMRM).

Table S2. Changes in RBC deformability and aggregation from baseline to weeks 4 and 12.

Parameter	Visit	Measurement	Experimental Group	Control Group	*p*-value	*p*-value‡	*Between-group difference (95% CI)*
RBC deformability (EI at 3 Pa) (%)	Visit 1	n (%)	31 (100.00)	30 (100.00)	0.4358**	0.4957	
		Mean±SD	0.33±0.02	0.33±0.01			
		Median	0.33	0.33			
		Min, Max	0.27, 0.36	0.30, 0.36			
	Visit 2	n (%)	31 (100.00)	30 (100.00)	0.3372**		
		Mean±SD	0.33±0.02	0.33±0.02			
		Median	0.33	0.34			
		Min, Max	0.30, 0.38	0.27, 0.36			
	Change (Visit 2 - Visit 1)	n (%)	0.00±0.02	0.00±0.02	0.5984*		
		Mean±SD	0.00	0.00			
		Median	-0.04, 0.04	-0.06, 0.04			
		Min, Max	0.6573†	0.7440†			
		p-value	0.6573†	0.7440†			
	Visit 3	n (%)	31 (100.00)	30 (100.00)	0.5637**		
		Mean±SD	0.32±0.03	0.33±0.02			
		Median	0.33	0.33			
		Min, Max	0.20,0.37	0.30,0.39			
	Change (Visit 3 - Visit 1)	n (%)	31 (100.00)	30 (100.00)	0.9253**		0.00 (−0.013 to 0.013)
		Mean±SD	0.00±0.03	0.00±0.02			
		Median	0.00	0.00			
		Min, Max	-0.13,0.05	-0.05,0.06			
		p-value	0.8646††	0.5935†			
Aggregation index (%)	Visit 1	n (%)	31 (100.00)	30 (100.00)	0.2217*	0.8388	
		Mean±SD	44.18±7.41	46.64±8.14			
		Median	45.44	47.59			
		Min, Max	30.32, 59.74	31.41, 61.79			
	Visit 2	n (%)	31 (100.00)	30 (100.00)	0.3072**		
		Mean±SD	43.58±6.82	45.66±8.91			

		Median	45.01	47.50			
		Min, Max	27.68, 53.10	21.47, 61.67			
	Change (Visit 2 - Visit 1)	n (%)	-0.61±7.23	-0.98±8.25	0.8518*		
		Mean±SD	-0.44	1.11			
		Median	-15.42, 13.52	-22.01, 17.38			
		Min, Max	0.6444†	0.5215†			
		p-value	0.6444†	0.5215†			
	Visit 3	n (%)	31 (100.00)	30 (100.00)	0.5637**		
		Mean±SD	43.64±8.37	47.15±9.74			
		Median	45.31	47.56			
		Min, Max	24.06,62.24	22.48,63.60			
	Change (Visit 3 - Visit 1)	n (%)	31 (100.00)	30 (100.00)	0.9253**		−1.04 (−5.77 to 3.69)
		Mean±SD	-0.54±7.67	0.50±10.49			
		Median	0.44	1.31			
		Min, Max	-18.24,21.09	-23.33,27.30			
		p-value	0.6961††	0.7948†			
Critical shear stress (Pa)	Visit 1	n (%)	31 (100.00)	30 (100.00)	0.1942**	0.5302	
		Mean±SD	361.81±205.53	359.04±113.10			
		Median	293.00	332.05			
		Min, Max	210.40, 1,241.90	199.70, 732.60			
	Visit 2	n (%)	31 (100.00)	30 (100.00)	0.0942**		
		Mean±SD	295.79±111.59	332.72±120.38			
		Median	260.70	302.10			
		Min, Max	144.80, 807.80	199.30, 781.20			
	Change (Visit 2 - Visit 1)	n (%)	31 (100.00)	30 (100.00)	0.5590**		
		Mean±SD	-66.05±212.07	-26.32±133.30			
		Median	-25.00	-17.80			
		Min, Max	-816.30, 469.70	-346.80, 282.60			
		p-value	0.0191†	0.2884†			
	Visit 3	n (%)	31 (100.00)	30 (100.00)	0.3672**		
		Mean±SD	331.72±135.72	330.92±80.25			
		Median	300.60	311.45			

		Min, Max	216.00,776.10	223.80,562.10			
	Change (Visit 3 - Visit 1)	n (%)	31 (100.00)	30 (100.00)			−1.95 (−93.72 to 89.82)
		Mean±SD	-30.11±222.23	-28.13±121.13			
		Median	2.20	-12.75			
		Min, Max	-899.20,475.80	-378.40,149.20			
		p-value	0.8334††	0.4378††			

* Independent t-test, ** Wilcoxon rank-sum test, †Paired t-test, ††Wilcoxon signed-rank test, ‡P values were obtained using a mixed-effects model for repeated measures (MMRM).

Table S3. Changes in FMD from baseline to weeks 4 and 12.

FMD (%)		Experimental Group	Control Group	*p*-value	*p*-value‡
Visit 1	n (%)	31 (100.00)	30 (100.00)	0.8966**	0.5644
	Mean±SD	3.95±3.65	3.43±1.87		
	Median	3.20	3.15		
	Min, Max	0.80, 19.00	0.70, 8.50		
Visit 2	n (%)	31 (100.00)	30 (100.00)	0.9770**	
	Mean±SD	4.05±2.20	4.11±2.23		
	Median	3.80	3.90		
	Min, Max	0.80, 11.40	0.90, 8.70		
Change (Visit 2 - Visit 1)	n (%)	31 (100.00)	30 (100.00)	0.4517**	
	Mean±SD	0.10±3.00	0.68±1.12		
	Median	0.20	0.60		
	Min, Max	-14.40, 4.70	-1.10, 3.20		
	p-value	0.0985††	0.0017††		
Visit 3	n (%)	31 (100.00)	30 (100.00)	0.8853**	
	Mean±SD	4.60±3.06	4.68±2.93		
	Median	3.80	4.15		
	Min, Max	0.80, 14.00	0.50, 11.40		
Change (Visit 3 – Visit 1)	n (%)	31 (100.00)	30 (100.00)	0.7727**	
	Mean±SD	0.65±2.98	1.25±2.68		
	Median	0.40	0.70		
	Min, Max	-10.10, 7.00	-3.10, 9.40		
	p-value	0.0467††	0.0173††		

** Wilcoxon rank-sum test, †paired t-test, ††Wilcoxon signed-rank test, ‡P values were obtained using a mixed-effects model for repeated measures (MMRM).

Table S4. Changes in the tODI from baseline to weeks 4 and 12.

**tODI**		**Experimental Group (N=31)**	**Control Group (N=30)**	***p*-value**	***p*-value‡**
Visit 1	Mean±SD	3.27±0.51	3.18±0.42	0.4841**	0.7224
	Median	3.31	3.19		
	Min, Max	2.45, 5.39	2.50, 4.13		
Visit 2	Mean±SD	3.26±0.35	3.23±0.28	0.7910*	
	Median	3.33	3.24		
	Min, Max	2.59, 4.00	2.46, 3.78		
Change (Visit 2-Visit 1)	Mean±SD	-0.02±0.52	0.05±0.48	0.5023**	
	Median	0.08	0.15		
	Min, Max	-1.95, 0.68	-1.18, 0.73		
	p-value	0.4538††	0.5379†		
Percent Change (Visit 2-Visit 1)	Mean±SD	0.90±13.59	3.25±14.62	0.5180*	
	Median	2.30	5.06		
	Min, Max	-36.15, 27.58	-32.33, 29.15		
Visit 3	Mean±SD	3.34±0.41	3.22±0.40	0.2710*	
	Median	3.37	3.17		
	Min, Max	2.43, 4.38	2.62, 4.30		
Change (Visit 3-Visit 1)	Mean±SD	0.07±0.75	0.04±0.46	0.7564**	
	Median	0.06	-0.02		
	Min, Max	-2.91, 1.66	-0.88, 0.78		
	p-value	0.2796††	0.6187†		
Percent Change (Visit 3-Visit 1)	Mean±SD	4.50±21.44	2.49±14.63	0.8682**	
	Median	1.92	-0.54		
	Min, Max	-53.94, 61.22	-24.11, 31.32		

* Independent t-test, ** Wilcoxon rank sum test, † Paired t-test†† Wilcoxon singed-rank test, ‡P values were obtained using a mixed-effects model for repeated measures (MMRM).

Percent change (%) = [(tODI at Week 4 or Week 12 - baseline tODI) / baseline tODI] × 100

Table S5. Changes in lipid profile from baseline to weeks 4 and 12.

Marker	Timepoint	Measure	Experimental Group (N=31)	Control Group (N=30)	*p*-value	*p*-value‡
Total Cholesterol	Visit 1	Mean±SD	159.81±29.66	156.00±49.93	0.4027**	0.3050
		Median	154.00	150.50		
		Min, Max	115.00, 210.00	88.00, 287.00		
	Visit 2	Mean±SD	157.74±32.92	159.40±37.56	0.8512**	
		Median	153.00	154.00		
		Min, Max	107.00, 236.00	107.00, 262.00		
	Change (Visit 2-Visit 1)	Mean±SD	-2.06±15.96	3.04±30.48	0.4297*	
		Median	-5.00	0.00		
		Min, Max	-34.00, 36.00	-61.00, 86.00		
		p-value	0.4769†	0.5807†		
	Percent Change (Visit 2-Visit 1)	Mean±SD	-1.21±9.33	5.59±26.80	0.5209**	
		Median	-2.61	0.81		
		Min, Max	-16.41, 18.00	-36.31, 97.73		
	Visit 3	Mean±SD	160.81±30.09	160.42±26.66	0.9655**	
		Median	161.00	160.00		
		Min, Max	103.00, 233.00	118.00, 277.00		
	Change (Visit 3-Visit 1)	Mean±SD	1.74±20.28	10.20±39.51	0.5022**	
		Median	0.00	3.00		
		Min, Max	-51.00, 38.00	-55.00, 97.00		
		p-value	0.4092†	0.2806†		
	Percent Change (Visit 3-Visit 1)	Mean±SD	0.11±12.71	10.29±30.03	0.4932**	
		Median	0.00	2.04		
		Min, Max	-33.12, 24.79	-29.73, 81.82		
HDL-C	Visit 1	Mean±SD	55.61±11.89	50.03±14.71	0.1120*	0.4738
		Median	54.00	48.00		
		Min, Max	39.00, 81.00	25.00, 84.00		

	Visit 2	Mean±SD	55.19±13.04	52.37±13.51	0.4055*	
		Median	52.00	51.00		
		Min, Max	36.00, 87.00	30.00, 80.00		
	Change (Visit 2-Visit 1)	Mean±SD	-0.35±7.34	2.34±8.95	0.2404*	
		Median	0.00	1.00		
		Min, Max	-20.00, 15.00	-16.00, 22.00		
		p-value	0.7897†	0.1604†		
	Percent Change (Visit 2-Visit 1)	Mean±SD	-0.26±12.57	7.92±23.12	0.1532**	
		Median	-0.08	1.61		
		Min, Max	-30.30, 28.85	-30.77, 88.00		
	Visit 3	Mean±SD	57.91±11.86	51.07±12.38	0.0205**	
		Median	55.35	50.00		
		Min, Max	39.00, 97.00	29.00, 70.00		
	Change (Visit 3-Visit 1)	Mean±SD	2.42±9.30	1.34±7.72	0.2506**	
		Median	2.00	1.00		
		Min, Max	-22.00, 20.00	-15.00, 22.00		
		p-value	0.1578†	0.6724†		
	Percent Change (Visit 3-Visit 1)	Mean±SD	5.96±16.52	5.48±23.53	0.2989**	
		Median	4.65	1.61		
		Min, Max	-30.40, 41.67	-16.67, 100.00		
LDL-C	Visit 1	Mean±SD	87.82±22.80	84.17±23.94	0.8033*	0.0892
		Median	83.40	81.00		
		Min, Max	35.00, 149.00	38.00, 159.00		
	Visit 2	Mean±SD	84.30±30.52	90.23±34.94	0.3090**	
		Median	80.00	84.00		
		Min, Max	50.00, 164.00	52.00, 200.00		
	Change (Visit 2-Visit 1)	Mean±SD	-0.03±14.44	7.27±31.53	0.2544*	
		Median	0.00	5.50		

		Min, Max	-46.00, 34.00	-57.00, 82.00		
		p-value	0.9902†	0.2161†		
	Percent Change (Visit 2-Visit 1)	Mean±SD	-0.03±14.44	7.16±46.09	0.2368**	
		Median	0.00	6.29		
		Min, Max	-30.87, 35.42	-48.11, 71.05		
	Visit 3	Mean±SD	85.55±26.08	95.47±32.12	0.4574**	
		Median	87.40	88.00		
		Min, Max	32.00, 141.00	55.00, 180.00		
	Change (Visit 3-Visit 1)	Mean±SD	-2.68±24.41	10.73±32.76	0.5254**	
		Median	4.00	3.00		
		Min, Max	-57.00, 80.00	-50.00, 92.00		
		p-value	0.2327†	0.2926†		
	Percent Change (Visit 3-Visit 1)	Mean±SD	-1.41±29.61	5.90±36.03	0.5935**	
		Median	2.09	1.61		
		Min, Max	-61.54, 95.15	-56.70, 85.00		

* Independent t-test, ** Wilcoxon rank-sum test, #Paired t-test, **Wilcoxon signed-rank test, ‡P values were obtained using a mixed-effects model for repeated measures (MMRM).

Table S6. Changes in fasting blood glucose levels from baseline to weeks 4 and 12.

Timepoint	Measure	Experimental Group (N=31)	Control Group (N=30)	*p*-value	*p*-value‡
Visit 1	Mean±SD	122.39±42.35	120.27±31.51	0.8624**	0.3828
	Median	111.00	110.00		
	Min, Max	76.00, 280.00	88.00, 214.00		
Visit 2	Mean±SD	120.32±38.18	107.80±17.03	0.2725**	
	Median	104.00	102.50		
	Min, Max	65.00, 236.00	87.00, 155.00		
Change (Visit 2-Visit 1)	Mean±SD	-2.06±33.93	-12.47±30.12	0.3555**	
	Median	-4.00	-5.50		
	Min, Max	-70.00, 87.00	-109.00, 32.00		
	p-value	0.5975††	0.0420†		
Percent Change (Visit 2-Visit 1)	Mean±SD	1.56±26.06	-6.83±18.80	0.3023**	
	Median	-3.70	-5.08		
	Min, Max	-48.41, 76.99	-52.91, 31.63		
Visit 3	Mean±SD	116.03±24.20	108.07±24.71	0.1867**	
	Median	106.00	101.00		
	Min, Max	87.00, 182.00	83.00, 221.00		
Change (Visit 3-Visit 1)	Mean±SD	-6.35±32.81	-12.20±37.66	0.4486**	
	Median	-5.00	-5.00		
	Min, Max	-117.00, 69.00	-111.00, 111.00		
	p-value	0.3064††	0.0209†		
Percent Change (Visit 3-Visit 1)	Mean±SD	-0.65±21.92	-6.25±26.15	0.3596**	
	Median	-5.00	-5.00		
	Min, Max	-41.79, 61.06	-51.87, 100.91		

** Wilcoxon rank-sum test, **Wilcoxon signed-rank test. ‡P values were obtained using a mixed-effects model for repeated measures (MMRM).

Table S7. Changes in HOMA-IR from baseline to weeks 4 and 12.

Timepoint	Measure	Experimental Group (N=31)	Control Group (N=30)	*p*-value	*p*-value‡
Visit 1	Mean±SD	4.49±5.01	7.52±14.56	0.8456**	0.2081
	Median	3.35	2.94		
	Min, Max	0.57, 25.27	0.65, 63.95		
Visit 2	Mean±SD	5.29±9.34	3.46±3.48	0.9368**	
	Median	2.38	2.18		
	Min, Max	0.63, 41.43	0.92, 18.56		
Change (Visit 2-Visit 1)	Mean±SD	0.80±10.43	-4.07±13.07	0.7455**	
	Median	-0.08	-0.50		
	Min, Max	-19.88, 37.25	-59.59, 4.32		
	p-value	0.3760††	0.1562†		
Percent Change (Visit 2-Visit 1)	Mean±SD	56.89±245.35	-2.88±56.72	0.7564**	
	Median	-1.61	-17.60		
	Min, Max	-88.81, 996.30	-93.18, 116.59		
Visit 3	Mean±SD	3.79±5.10	4.51±9.64	0.8119**	
	Median	2.01	2.50		
	Min, Max	0.43, 25.84	0.70, 54.02		
Change (Visit 3-Visit 1)	Mean±SD	-0.70±6.56	-3.02±17.63	0.7130**	
	Median	-0.78	-0.50		
	Min, Max	-23.23, 21.66	-60.48, 51.96		
	p-value	0.1871††	0.0528†		
Percent Change (Visit 3-Visit 1)	Mean±SD	18.88±132.83	75.00±468.18	0.8910**	
	Median	-24.00	-24.77		
	Min, Max	-91.91, 517.44	-94.57, 2,527.43		

(** Wilcoxon rank-sum test, **Wilcoxon signed-rank test, ‡P values were obtained using a mixed-effects model for repeated measures (MMRM). #HOMA-IR = (fasting insulin × fasting blood glucose) / 405).

Table S8. Changes in hs-CRP levels from baseline to weeks 4 and 12.

**Timepoint**	**Measure**	**Experimental Group (N=31)**	**Control Group (N=30)**	***p*-value**	***p*-value‡**
Visit 1	Mean±SD	0.13±0.13	0.30±0.62	0.1602**	0.4330
	Median	0.06	0.11		
	Min, Max	0.06, 0.58	0.06, 3.38		
Visit 2	Mean±SD	0.12±0.15	0.18±0.28	0.6330**	
	Median	0.06	0.11		
	Min, Max	0.06, 0.68	0.06, 1.30		
Change (Visit 2-Visit 1)	Mean±SD	-0.01±0.08	-0.12±0.62	0.2744**	
	Median	0.00	0.00		
	Min, Max	-0.25, 0.18	-2.79, 1.24		
	p-value	0.3814††	0.1108†		
Percent Change (Visit 2-Visit 1)	Mean±SD	-0.20±63.49	84.17±429.77	0.3020**	
	Median	0.00	0.00		
	Min, Max	-75.76, 300.00	-92.73, 2,066.67		
Visit 3	Mean±SD	0.16±0.19	0.17±0.29	0.8852**	
	Median	0.07	0.07		
	Min, Max	0.06, 0.78	0.06, 1.51		
Change (Visit 3-Visit 1)	Mean±SD	0.02±0.14	-0.13±0.66	0.0250**	
	Median	0.00	-0.01		
	Min, Max	-0.20, 0.54	-3.11, 1.30		
	p-value	0.7893††	0.0351††		
Percent Change (Visit 3-Visit 1)	Mean±SD	24.50±104.47	8.33±138.64	0.0265**	
	Median	0.00	-4.55		
	Min, Max	-70.83, 490.91	-94.55, 619.05		

** Wilcoxon rank-sum test, **Wilcoxon signed-rank test. , ‡P values were obtained using a mixed-effects model for repeated measures (MMRM).

Table S9. Adverse Reaction Incidence Classified by Organ System

Classified by System Organ Class †	Experimental group N=34 n(%) [cases]††	Control group N=34 n(%) [cases]††	Total N=68 n(%) [cases]††
Subjects with AEs	9(26.47) [9]	8(23.53) [9]	18(26.47) [18]
**Gastrointestinal disorders**	**3(8.82) [3]**	**1(2.94) [1]**	**4(5.88) [4]**
Gastrooesophageal reflux disease	1(2.94) [1]	0(0.00) [0]	1(1.47) [1]
Gingival bleeding	1(2.94) [1]	1(2.94) [1]	2(2.94) [2]
Haematochezia	1(2.94) [1]	0(0.00) [0]	1(1.47) [1]
**General disorders and administration site conditions**	**0(0.00) [0]**	**1(2.94) [1]**	**1(1.47) [1]**
Gait disturbance	0(0.00) [0]	1(2.94) [1]	1(1.47) [1]
**Hepatobiliary disorders**	**1(2.94) [1]**	**0(0.00) [0]**	**1(1.47) [1]**
Hepatitis	1(2.94) [1]	0(0.00) [0]	1(1.47) [1]
**Infections and infestations**	**0(0.00) [0]**	**1(2.94) [1]**	**1(1.47) [1]**
Cystitis	0(0.00) [0]	1(2.94) [1]	1(1.47) [1]
**Investigations**	**1(2.94) [1]**	**0(0.00) [0]**	**1(1.47) [1]**
SARS-CoV-2 test positive	1(2.94) [1]	0(0.00) [0]	1(1.47) [1]
**Nervous system disorders**	**2(5.88) [2]**	**2(5.88) [2]**	**4(5.88) [4]**
Headache	1(2.94) [1]	0(0.00) [0]	1(1.47) [1]
Paraesthesia	1(2.94) [1]	1(2.94) [1]	2(2.94) [2]
Vertigo CNS origin	0(0.00) [0]	1(2.94) [1]	1(1.47) [1]
**Renal and urinary disorders**	**0(0.00) [0]**	**2(5.88) [2]**	**2(2.94) [2]**
Haematuria	0(0.00) [0]	1(2.94) [1]	1(1.47) [1]
Proteinuria	0(0.00) [0]	1(2.94) [1]	1(1.47) [1]
**Respiratory, thoracic and mediastinal disorders**	**2(5.88) [2]**	**1(2.94) [1]**	**3(4.41) [3]**
Epistaxis	0(0.00) [0]	1(2.94) [1]	1(1.47) [1]
Haemoptysis	1(2.94) [1]	0(0.00) [0]	1(1.47) [1]
Oropharyngeal pain	1(2.94) [1]	0(0.00) [0]	1(1.47) [1]
**Skin and subcutaneous tissue disorders**	**0(0.00) [0]**	**1(2.94) [1]**	**1(1.47) [1]**
Urticaria chronic	0(0.00) [0]	1(2.94) [1]	1(1.47) [1]

† Coding dictionary: MedDRA Version 27.0

†† Double counted
